# Oxidative Decarboxylation of Levulinic Acid by Silver(I)/Persulfate

**DOI:** 10.3390/molecules16032714

**Published:** 2011-03-23

**Authors:** Yan Gong, Lu Lin

**Affiliations:** Department of Resources Science and Engineering, State Key Lab of Pulp and Paper Engineering, South China University of Technology, Guangzhou 510640, China

**Keywords:** oxidative decarboxylation, levulinic acid, silver nitrate, potassium persulfate

## Abstract

The oxidative decarboxylation of levulinic acid (LA) by silver(I)/persulfate [Ag(I)/S_2_O_8_^2−^] has been investigated in this paper. The effects of buffer solution, initial pH value, time and temperature and dosages of Ag(I)/S_2_O_8_^2−^ on the decarboxylation of LA were examined in batch experiments and a reaction scheme was proposed on basis of the reaction process. The experimental results showed that a solution of NaOH-KH_2_PO_4 _was comparatively suitable for the LA decarboxylation reaction by silver(I)/persulfate. Under optimum conditions (temperature 160 °C, pH 5.0, and time 0.5 h), the rate of LA conversion in NaOH-KH_2_PO_4 _solutions with an initial concentration of 0.01 mol LA reached 70.2%, 2-butanone (methyl ethyl ketone) was the single product in the gas phase and the resulted molar yield reached 44.2%.

## 1. Introduction

Levulinic acid (4-oxo-pentanoic acid), results from a series of biomass hydrolysis reactions [1,2]. With its wide application profile it is an important chemical platform obtainable from renewable lignocelluloses [3,4,5] because of its molecular structure that contains a carbonyl group and a carboxyl group. Hydrogenation of levulinic acid, can produce γ-valerolactone [6], a potentially useful polyester monomer (as γ-hydroxyvaleric acid); 1,4-pentanediol, also of value in polyester production; methyltetrahydrofuran, a valuable solvent or a gasoline blending component; and diphenolic acid with potential for use in polycarbonate production [7] are other significant derivatives. 

Decarboxylation of organic molecules happens by the removal of a carboxyl group from the carboxylic acids, further replaced by a hydrogen atom. Up to date, oxidative decarboxylation with various agents have been repeatedly reported as the most common pathway for the decarboxylation of carboxylic acids and their derivatives, and a variety of oxidants involved in these reactions have been extensively studied, such as porphyrin combined with manganese (Ш) [8], Schiff base complexes combined with manganese (III) [9], active hydroxyl radicals (•OH) [10], peroxyl radicals [11], and high-valent metal ions [12,13,14]. As for decarboxylation of LA, to the best of our knowledge until now only two reports exist; one is for the photoelectron-chemical decarboxylation of LA [15], leading to 2-butanone, propionic acid, acetic acid, acetone and acetaldehyde as major products, but the yield of butanone was not satisfactory. Another is for oxidative decarboxylation of LA catalyzed by cupric oxide (CuO) [16] which came from our laboratory. As for 2-butanone, it is a product of LA decarboxylation, which can be directly hydrogenated to butanol [17,18] or be converted to ethyl acetate through Baeyer-Villiger oxidation [19] and further reduced to ethanol. Therefore, decarboxylation of levulinic acid to produce butanone and its consequent product of ethanol could play an important role to meet future energy needs and solve environmental problems caused by fossil fuels.

In the previous report of our laboratory on oxidative decarboxylation of LA [16], it was found that the yield of butanone can reach 67% (mol ratio), and acetic acid and acetone were also produced as side products. However, the reaction conditions used were extremely drastic (temperature was over 300 °C). In this paper, a novel pathway for decarboxylation of LA by Ag(I)/S_2_O_8_^2− ^under mild conditions was researched. Persulfate anion (S_2_O_8_^2−^) is a strong oxidizing agent with a redox potential (Eo) of 2.01 V and its reduction results in the production of sulfate anions [20]. It is used in many fields such as oxidation of trichloroethylene (TCE) from groundwater contaminants [21], degradation of organic pollutants in wastewater [22]; oxidation–dealkylation of 3,4-dihydropyrimidin-2(1*H*)-ones in the presence of Co(NO_3_)_2_ [23]. Persulfate anion is also applied in the decarboxylation of carboxylic acids with or without various transition metal catalysts [24,25,26,27], et al*.* Therefore, utilization of persulfate anion for the decarboxylation of LA under milder conditions could open a new venue for prospective application of this reaction and its products in the future.

## 2. Results and Discussion

### 2.1. Decarboxylation of Levulinic Acid to 2-Butanone in Different Buffer Solutions

**Figure 1:**
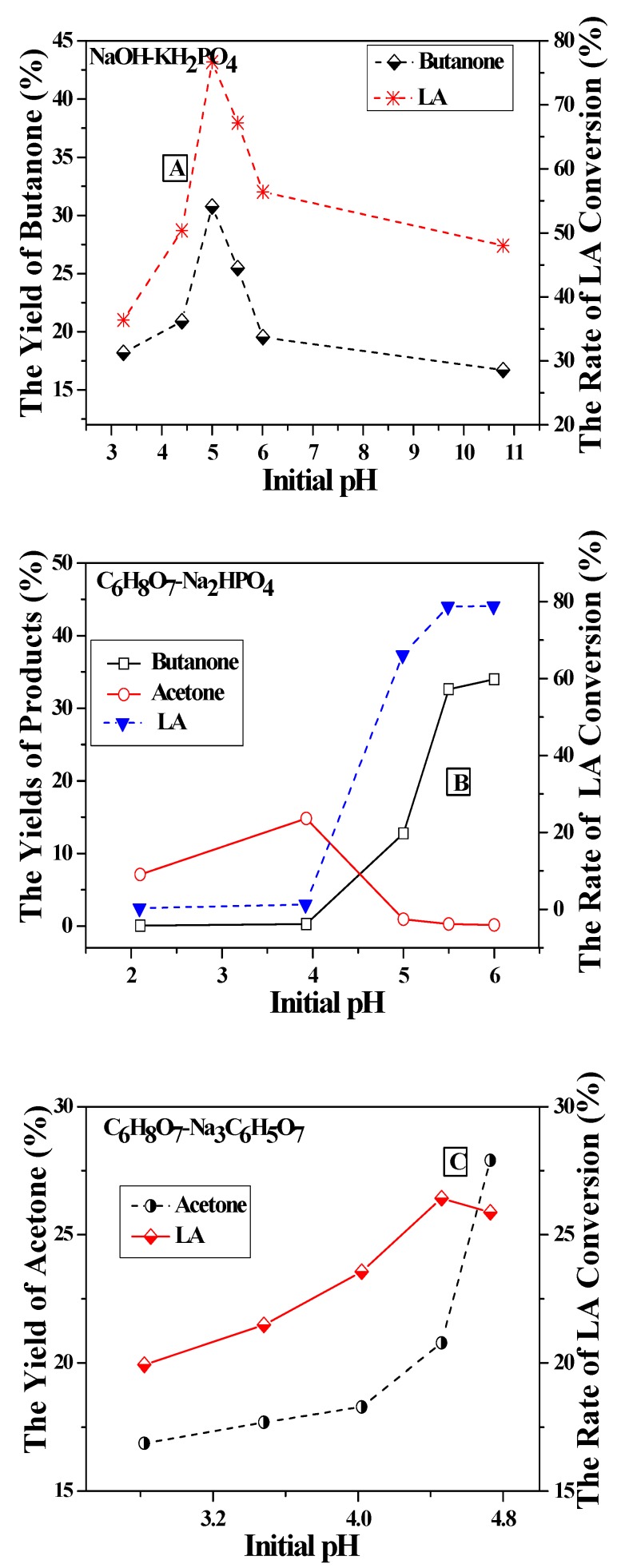
Effect of initial pH on the decarboxylation of levulinic acid in different buffer solutions (A: NaOH-KH_2_PO_4_; B: C_6_H_8_O_7_-Na_2_HPO_4_; C: C_6_H_8_O_7_-Na_3_C_6_H_5_O_7_; temperature: 100 °C; time: 0.5 h).

The experiments of LA decarboxylation by Ag(I)/ S_2_O_8_^2−^ were carried out at 100 °C for 30 min in an oil bath. From Figure 1A, it could be found that in NaOH-KH_2_PO_4_ solution, as pH values increased, both the rate of LA conversion and yield of butanone increased and reached about 30% and 43%, respectively, at pH 5.0, but then they decreased with further pH value increases. In C_6_H_8_O_7_-Na_2_HPO_4_ solution, as shown in Figure 1B, the rate of LA conversion and the yield of butanone went up with the pH values ranging from 2.1 to 6.0. Besides 2-butanone, acetone was also produced. At pH 2.1 and 3.9, the yields of acetone were higher than the rate of LA conversions. It was suggested that citric acid in the solution reacted with Ag(I)/ S_2_O_8_^2−^ and caused the production of acetone. Moreover, it was shown in Figure 1C that the only product in the gas phase was acetone. These results indicated that NaOH-KH_2_PO_4_ solution was comparatively suitable for LA decarboxylation by Ag(I)/ S_2_O_8_^2−^ to produce butanone.

### 2.2. Decarboxylation of Levulinic Acid to 2-Butanone under Different Times and Temperatures

**Figure 2:**
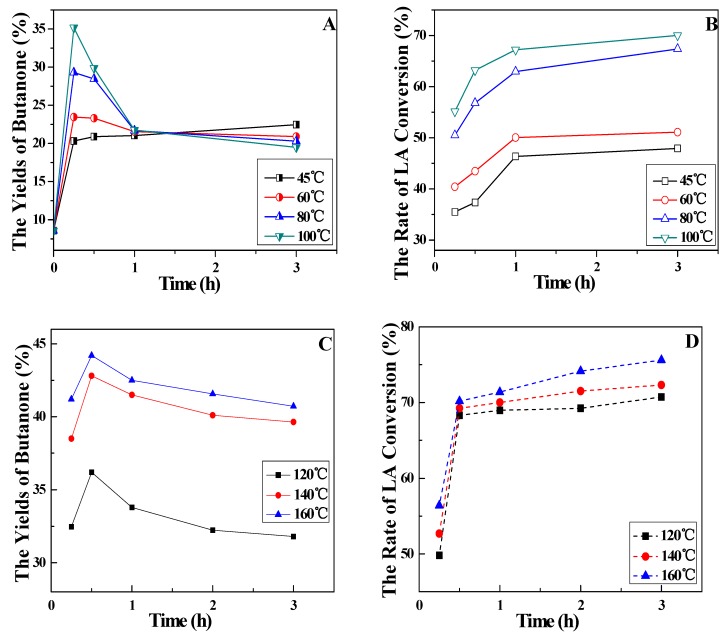
Effect of time on decarboxylation of levulinic acid to 2-butanone {[A] butanone yield (≤100 °C); [B] LA conversion(≤100 °C); [C] butanone yield (≥100 °C); [D] LA conversion (≥100 °C)}.

Based on the above results, the effect of reaction time at different temperatures on LA decarboxylation using Ag(I)/S_2_O_8_^2−^ in NaOH-KH_2_PO_4_ solution was further investigated. The experiments were carried out at lower temperatures (≤100 °C) in an oil bath. The results obtained from the experiments are shown in Figure 2A. It was found that the yields of butanone all increased rapidly within 15 min. After that, the yield of butanone at 45 °C still rose slightly, while it decreased at 60 °C, 80 °C and 100 °C. The highest yield of butanone was 35.2% at 100 °C for 15 min. However, the rate of LA conversions increased during the 3 h but the ascending trends were diminished after 1 h (Figure 2B).It was claimed that butanone from the LA decarboxylation also involved in the oxidation with Ag(I)/S_2_O_8_^2−^ and was disjoined and converted to other materials with smaller molecule. Besides, the oxidation of butanone to a certain extent inhibited the decarboxylation of LA.

When reactions were carried out in the pressure reactor at the temperature of 120 °C, 140 °C and 160 °C, it is shown in Figure 2C and Figure 2D that the yields of butanone were apparently improved within 0.5 h and reached 36.2%, 42.8% and 44.2%, respectively. With the increase of time, the yields of butanone all had a mild decline. The rate of LA conversions went up sharply in 0.5 h and then rose slowly for the residual reaction time, but LA was not converted completed finally after 3 h. Compared with the effectiveness in oil bath, it was better in autoclave, that is, the yields of butanone resulted from LA decarboxylation in autoclave were higher, at the same time, optimum time was prolonged to 0.5 h. We proposed that butanone vaporized at higher temperatures and was separated from the react solution, which reduced the contact between butanone and Ag(I)/ S_2_O_8_^2−^. 

**Figure 3:**
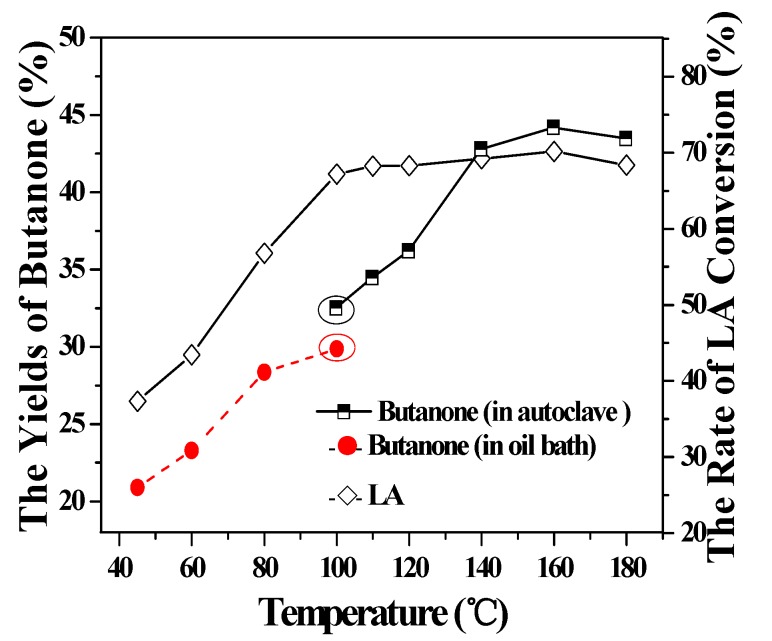
Effect of temperature to butanone yield (NaOH-KH_2_PO_4_; pH: 5.0; time: 0.5 h).

The effect of temperature from 45 °C to 180 °C in a time of 0.5 h on LA decarboxylation by Ag(I)/S_2_O_8_^2−^ is shown in Figure 3. The yields of butanone and the rate of LA conversions were gradually improved with the enhancement of temperature. The yield of butanone reached about 43% at 140 °C and then remained stable as temperature rose, while the rate of LA conversion reached 67.2% at 100 °C and had no distinct change when higher temperatures were applied. At the temperature of 100 °C, the experiments were performed both in oil bath and autoclave and the yields of butanone were 29.9% and 32.6%, respectively. It is presumed that this resulted from the fact that the autoclave is a close system and therefore butanone loss was reduced and reaction efficiency was comparatively higher. Moreover, mechanical agitation in autoclave led to better mass and heat transfer for the reaction, which diminished side effects.

### 2.3. Decarboxylation of Levulinic Acid to 2-Butanone under Different Ratios of Substrate and Oxidant

As seen in Table 1, when LA was oxidatively decarboxylated by S_2_O_8_^2−^ without Ag(I) under the same conditions, the rate of LA conversion and the yield of butanone were similar with those from LA decarboxylation by Ag(I)/S_2_O_8_^2−^.

**Figure 4:**
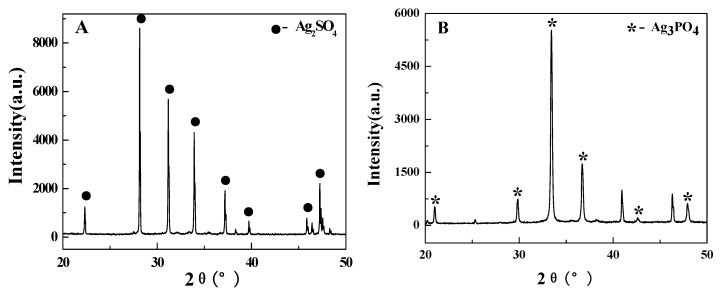
The X-ray diffraction spectra of the reaction residues [A: Ag(I)/S_2_O_8_^2−^; B: Ag(I)].

In contrast, reaction of Ag(I) with LA without S_2_O_8_^2−^ led to production of butanone at a low efficiency (LA conversion 12.9%; butanone yield 1.2%). The XRD analyses of the final residues are shown in Figure 4, where Figure 4A and Figure 4B are the XRD spectra of silver sulfate (Ag_2_SO_4_) and silver phosphate (Ag_3_PO_4_), respectively, which corresponded to the residues from the decarboxylation of LA by Ag(I)/S_2_O_8_^2−^ and Ag(I). The ratios of LA with Ag(I)/S_2_O_8_^2−^ ranging from 0.5 to 1.5 were investigated, indicating that the efficiency of decarboxylation went up as the ratio was increased and it was relatively lower at the ratio of 0.5, but less change was found at other ratios.

**Table 1:** Effect of ratio of substrate and oxidant on the yield of butanone and the rate of LA conversion (temperature: 120 °C; time: 0.5 h; pH: 5.0).

Metal ion catalysts	Yield of butanone (%)	The Rate of LA conversion (%)
Ag(I)	1.2	12.9
S_2_O_8_^2-^	35.9	69.3
0.5:1 Ag(I)/S_2_O_8_^2-^	28.8	34.8
1:1 Ag(I)/S_2_O_8_^2-^	36.2	69
1.2:1 Ag(I)/S_2_O_8_^2-^	36.4	70
1.5:1 Ag(I)/S_2_O_8_^2-^	36.7	72

### 2.4. Decarboxylation of Levulinic Acid to 2-Butanone under Different Metal Ion Catalysts

Decarboxylation of LA by other metal ions combined with S_2_O_8_^2−^ was studied further. The experiments were carried out in a DF-101S oil bath at 100 °C for 0.5 h. It is shown in Table 2 that Ag(I)/S_2_O_8_^2−^ was the most effective agent for LA decarboxylation to butanone, followed by Co(II)/S_2_O_8_^2−^. The yields of butanone were lower with Fe(II)/S_2_O_8_^2−^ and Mn(II)/S_2_O_8_^2−^. However, when LA reacted with Cu(I)/S_2_O_8_^2−^, it was shown from the results HS-GC-MS analysis that methyl vinyl ketone was produced as the main gaseous product (about 10% by HS-GC), other products included 2,3-butanedione and 2-chloro-1-buten-3-one. Almost no product could be detected by HS-GC in the reaction system of LA and Cu(II)/S_2_O_8_^2−^.

**Table 2:** Effect of metal ions on the yield of butanone and the rate of LA conversion in S_2_O_8_^2−^ reaction system (temperature: 100 °C; time: 0.5 h; pH: 5.0).

Metal ion catalysts	Yield of butanone /%	Rate of LA conversion/%
Ag(I)^ a^ /S_2_O_8_^2-^	35.2	67.2
Co(II)^ b^ / S_2_O_8_^2-^	6.63	20.73
Cu(I)^ c^ / S_2_O_8_^2-^	—	25.42
Cu(II)^ d^ / S_2_O_8_^2-^	—	20.36
Fe(II)^ e^ / S_2_O_8_^2-^	1.1	92.6
Mn(II)^ f^ / S_2_O_8_^2-^	5.91	77.42

^a^ AgNO_3_; ^b^ Co(NO_3_)_2_; ^c^ CuCl; ^d^ CuCl_2_; ^e ^FeCl_2_; ^f^ MnCl_2_

### 2.5. Reaction scheme for LA decarboxylation by Ag(I)/S_2_O_8_^2−^

**Figure 5:**
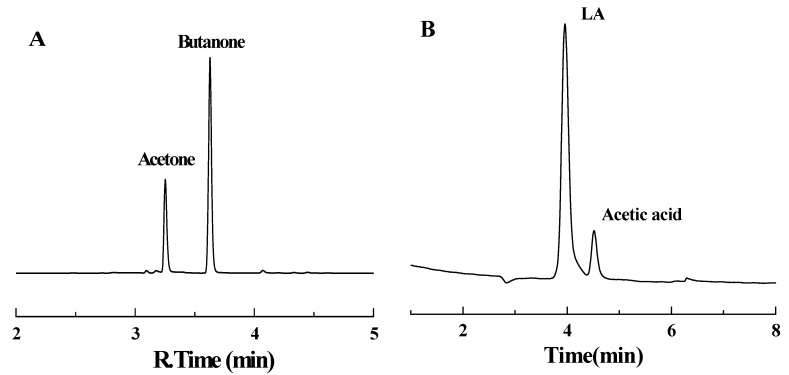
Headspace - gas chromatogram (A) and Ion Chromatogram (B) of samples.

**Figure 6:**
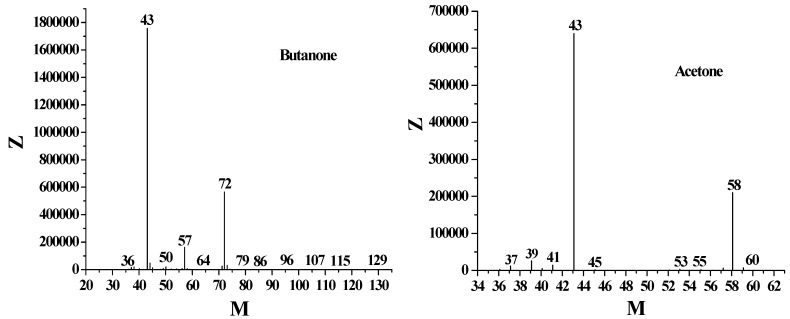
Mass spectrograms of butanone and acetone.


A mechanism for the oxidative decarboxylation of aliphatic carboxylic acids by the Ag(I)/S_2_O_8_^2-^ system has been described by Anderson *et al.* [28]. According to the HS-GC/GC-MS experimental analysis shown in Figure 5A and Figure 6 and the ion chromatography data in Figure 5B, LA undergoes oxidative decarboxylation by Ag(II) ion which can be produced by persulfate oxidation of Ag(I) as shown in equations (1–5). In this scheme, the oxidation of Ag(I) by persulfate (eq. 1) is followed in quick succession by a second oxidation by the sulfate ion radical (eq. 2), in accordance with the kinetic study of the persulfate oxidation demonstrated by Miller [29]. Direct and facile oxidation of LA by Ag(II) in a fast follow-up step could bring about an anion of CH_3_COCH_2_CH_2_CO_2_·, which could be subsequently fragmented to an CH_3_COCH_2_CH_2_· and CO_2_. Butanone was derived by oxidation of the CH_3_COCH_2_CH_2_• with silver species as well as by hydrogen transfer to solvent (eqs. 3–5). It was said that the activation process for Ag(I)-catalyzed oxidative decarboxylation by persulfate was largely associated with the formation of Ag(II) species [28], but it appears from Table 1 that the yield of butanone from S_2_O_8_^2−^ oxidation is 35.9%, near to 36.2% which comes from Ag(I)/S_2_O_8_^2−^ oxidation. It is reasonable to assume that S_2_O_8_^2−^ can oxidize LA independently. We propose the mechanism of S_2_O_8_^2−^ reacting with LA without Ag(I) in eqs. (6–7) that sulfate ion radical (SO_4_^−^·) is from the thermal decomposition of S_2_O_8_^2−^, and it can directly oxidize LA.


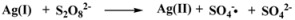
(1)


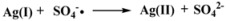
(2)


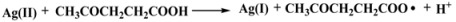
(3)


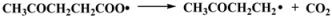
(4)


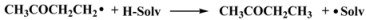
(5)


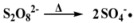
(6)


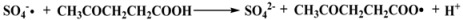
(7)

Selective oxidation of arylacetic acids in the presence of Cu(II)/S_2_O_8_^2−^ oxidation system has been reported [30] and the corresponding benzylacetates can be generated. It is conjectured that Cu(II)/S_2_O_8_^2−^ may oxidize LA to a kind of ester. The identification of methyl vinyl ketone arising from LA decarboxylation by Cu(I)/S_2_O_8_^2−^ oxidation indicated that eqs. (8–10) were operative. Oxidation of LA to alkene can also occur since the second-order rate constant of 10^8^–10^9 ^M^−1^ sec^−1^ for reaction (11) has been assigned according to the report by Anderson [28].

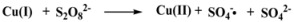
(8)


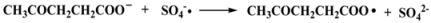
(9)


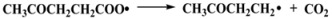
(10)


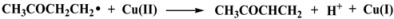
(11)


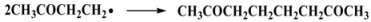
(12)

Benzylic radicals can be produced from the oxidative decarboxylation of arylacetic acids such as phenylacetic acid and dimerized further to 1,2-diarylethanes [31], thus, it is deduced that alkyl radical (CH_3_COCH_2_CH_2_) can dimerize to 2,7- dioctanone (eq. 12) in the liquid phase. However, further studies for a delicate exploration of this reaction procedure are required.

## 3. Experimental

### 3.1. Procedure for Decarboxylation of Levulinic Acid

Batch experiments were performed in a DF-101S oil bath (Yu Hua instrument), and a PARR-5050 Pressure Reactor (100 mL, Parr Instrument). Reaction solutions were obtained by adding levulinic acid (0.01 mol) into a buffer solution and then silver nitrate (0.01 mol) and potassium persulfate (0.005 mol) were added to the prepared reaction mixtures. After reaction, the samples were taken for HS-GC and ion chromatography detection. The final residues were analyzed by XRD after washing and desiccation. All experiments were repeated several times and shown to be reproducible.

### 3.2. Headspace – gas chromatography detection

Headspace - gas chromatography (HS-GC, HSS 86.50, QP2010, Shimadzu Japan) was used extensively in the separation and identification of mixtures of volatile compounds. The volatile products of LA decarboxylation were analyzed by HS-GC using a DB-5 column (30 m × 0.25 mm i.d. × 0.25 μm film) at a column temperature of 120 °C, a flow rate of 4.7 mL/min and with a flame ionization detector at the temperature of 220 °C. The analysis was performed at 60 °C for 5-min. The component concentrations were calculated by the area normalization method. The sample vials was shaken for 20 min at the temperature of 75 °C. The temperatures of sampling probe and tube were 85 °C and 95 °C; respectively. Automatic sampling was employed. The headspace - gas chromatogram of volatile products in samples (from C_6_H_8_O_7_-Na_2_HPO_4_ solution) was shown in Figure 5A that acetone and butanone were the gas products and the retention times were about 3.26 min and 3.63 min, respectively.

### 3.3. Ion chromatography detection

Ion chromatography (ICS-3000, DIONEX USA) was used for the analysis of levulinic acid in the aqueous phase to figure out the levulinic acid conversion. Liquid reactant after the reactions was filtered and diluted before testing. The analysis was conducted in an anion-exchange column (AS11-HC) with 2 mol/L NaOH leacheate in a conductivity cell of 35 °C. The flow rate was 1.0 mL/min and the sample size was 50 μL. The calibration was performed using aqueous solutions. Ion Chromatogram of samples was shown in Figure 5B that the retention time of levulinic acid was 4.06 min.

### 3.4. X-ray diffraction analysis

The precipitate after reactions was characterized by X-ray diffraction (XRD) (D/max-IIIA, Rigaku, Japan) (40 kV, 40 mA) using the standard reflection mode with Cu/Ka (λ = 0.154056 nm) in the angle 30° < 2θ < 90° and step angle was 0.02°.

## 4. Conclusions

It was shown that LA could be oxidatively decarboxylated by S_2_O_8_^2−^ with or without Ag(I) and leading to 2-butanone as the main gas product. When Ag(I) was replaced with Cu(I), the main product changed to methyl vinyl ketone. The conclusions from the reaction process are as follows: 2-butanone can be produced from biomass-derived LA, which is a matter of considerable interest for fuel sources and environmental problems; when the reaction was performed in NaOH-KH_2_PO_4_ solution with a pH value of 5.0 at a temperature of 160 °C and lasted for 0.5 h, the yield of butanone and the rate of LA conversion could reach 44.2% and 70.2%, respectively. Although the yield is lower than that obtained with CuO oxidative decarboxylation of LA (yield 67%), the reaction conditions are milder with shorter react time.
